# Longitudinal association between cognitive depressive symptoms and D‐dimer levels in patients following acute myocardial infarction

**DOI:** 10.1002/clc.23689

**Published:** 2021-07-07

**Authors:** Roland von Känel, Aju P. Pazhenkottil, Rebecca E. Meister‐Langraf, Hansjörg Znoj, Jean‐Paul Schmid, Claudia Zuccarella‐Hackl, Jürgen Barth, Ulrich Schnyder, Mary Princip

**Affiliations:** ^1^ Department of Consultation‐Liaison Psychiatry and Psychosomatic Medicine University Hospital Zurich, University of Zurich Zurich Switzerland; ^2^ Department of Cardiology University Hospital Zurich, University of Zurich Zurich Switzerland; ^3^ Cardiac Imaging, Department of Nuclear Medicine University Hospital Zurich, University of Zurich Zurich Switzerland; ^4^ Department of Psychiatry Clienia Schlössli AG, Oetwil am See Zurich Switzerland; ^5^ Department of Health Psychology and Behavioral Medicine University of Bern Bern Switzerland; ^6^ Department of Cardiology Clinic Barmelweid Barmelweid Switzerland; ^7^ Institute for Complementary and Integrative Medicine University Hospital Zurich, University of Zurich Zurich Switzerland; ^8^ Medical Faculty University of Zurich Zurich Switzerland

**Keywords:** coronary heart disease, coagulation, cortisol, psychological stress

## Abstract

**Background:**

A prothrombotic tendency could partially explain the poor prognosis of patients with coronary heart disease and depression. We hypothesized that cognitive depressive symptoms are positively associated with the coagulation activation marker D‐dimer throughout the first year after myocardial infarction (MI).

**Methods:**

Patients with acute MI (mean age 60 years, 85% men) were investigated at hospital admission (*n* = 190), 3 months (*n* = 154) and 12 months (*n* = 106). Random linear mixed regression models were used to evaluate the relation between cognitive depressive symptoms, assessed with the Beck depression inventory (BDI), and changes in plasma D‐dimer levels. Demographics, cardiac disease severity, medical comorbidity, depression history, medication, health behaviors, and stress hormones were considered for analyses.

**Results:**

The prevalence of clinical depressive symptoms (13‐item BDI score ≥ 6) was 13.2% at admission and stable across time. Both continuous (*p* < .05) and categorical (*p* < .010) cognitive depressive symptoms were related to higher D‐dimer levels over time, independent of covariates. Indicating clinical relevance, D‐dimer was 73 ng/ml higher in patients with a BDI score ≥ 6 versus those with a score < 6. There was a cognitive depressive symptom‐by‐cortisol interaction (*p* < .05) with a positive association between cognitive depressive symptoms and D‐dimer when cortisol levels were high (*p* < .010), but not when cortisol levels were low (*p* > .05). Fluctuations (up and down) of cognitive depressive symptoms and D‐dimer from one investigation to the next showed also significant associations (*p* < .05).

**Conclusions:**

Cognitive depressive symptoms were independently associated with hypercoagulability in patients up to 1 year after MI. Hypothalamic–pituitary–adrenal axis could potentially modify this effect.

## 1 INTRODUCTION

Psychosocial factors have been associated with an increased risk of cardiovascular disease (CVD), independently of and similarly strong as traditional biological risk factors.^1^ Depression is arguably the most reliably identified psychosocial risk factor of incident coronary heart disease (CHD)^2^ and poor prognosis after myocardial infarction (MI).^3^ Moreover, a recent meta‐analysis showed depression to be associated with an increased risk of major adverse cardiovascular events and all‐cause mortality in patients with CHD who underwent percutaneous coronary intervention.^4^ The pooled prevalence of depression among patients with MI is 29%, which is 2.5‐fold higher than in the general population.^5^ Even mild depressive symptoms may increase mortality risk, indicating a dose–response relation between depression severity and poor prognosis.^6^ The mechanisms contributing to adverse cardiovascular outcomes in patents with CHD and depression are multifactorial and incompletely understood.^7^

A clotting diathesis, including platelet hyperactivity, is one candidate mechanism for atherothrombotic complications in patients with CHD and depression.^7^, ^8^ However, the full sequence of events from acute MI to depression to a procoagulant state and thrombotic outcomes has not yet been demonstrated.^6^ To advance this knowledge, longitudinal investigations with reliable, easy‐to‐determine global hypercoagulability markers seem promising. For instance, the fibrin degradation product D‐dimer has a plasma half‐life of 8 h and indicates enhanced in vivo fibrin formation and coagulation activation with therapeutic and prognostic implications for the clinical management of a number of diseases.^9^ In experimental and observational studies, high D‐dimer levels were associated with both psychological distress^8^, ^10^ and CHD risk, independently of conventional risk factors.^11^ A meta‐analysis of 18 prospective population‐based studies with a total of 6799 individuals showed an association between D‐dimer and incident CHD risk after 2–19 years of follow‐up.^12^ Studies in patients referred for coronary intervention showed D‐dimer at admission to be a predictor of all‐cause mortality at 6 months^13^ and after 6.6 years.^14^ D‐dimer measured 2 months after MI was associated with an increased risk of recurrent coronary events 2 years later.^15^ In a study with 7863 patients, D‐dimer measured between 5 and 38 months after an acute coronary syndrome was predictive of an increased risk of major coronary or cardiovascular events after 6 years and with all‐cause and cardiovascular mortality after 16 years.^16^

We investigated the hypothesis of a longitudinal association between greater severity of cognitive depressive symptoms and higher plasma D‐dimer concentration in patients following MI investigated three times over 12 months. Using the Beck depression inventory (BDI), we examined the effect of cognitive depressive symptoms, because the prognostic impact of somatic symptoms of depression on cardiovascular outcomes is partially explained by cardiac disease severity.^17^ Moreover, compared with cognitive depressive symptoms, somatic ones such as fatigue show greater overlap with cardiac disease and may be a direct consequence of the cardiac disease.^17^ In addition to adjusting for cardiac health status and inflammation, we considered demographics, medical comorbidity, health behaviors and medication as potential confounding variables of D‐dimer levels.^18^ We also adjusted for a history of depression because first‐ever depressive symptoms after cardiac events seem particularly predictive of adverse outcomes.^19^ Finally, we examined whether norepinephrine and cortisol levels partially explain the relation between depressed mood and D‐dimer, as both hormones may link depression with CHD^20^ and are associated with hypercoagulability.^8^

## 2 METHODS

This was a secondary analysis of data collected from 190 patients who participated in the MI‐Stress Prevention Intervention (MI‐SPRINT) randomized controlled trial (RCT).^21^ The trial investigated whether one session of trauma‐focused counseling compared to stress counseling (active control condition), both delivered within 48 h after hospital admission, would prevent the development of MI‐induced posttraumatic stress at 3 months, which was not the case.^22^ The ethics committee of the State of Bern approved the study protocol, which was registered under ClinicalTrials.gov (NCT01781247). All participants provided written informed consent.

Inclusion criteria were a minimum age of 18 years, verified acute ST segment elevation MI (STEMI) or non‐STEMI, stable circulatory conditions and high distress during MI, defined by a minimum score of 5 (range 0–10) for pain intensity and fear of dying and/or helplessness. Exclusion criteria were emergency coronary artery bypass grafting, severe comorbid diseases, limited orientation, cognitive impairment, current severe depression (per the cardiologist's medical history), suicidal ideations in the prior 2 weeks, insufficient German skills, and participation in another randomized controlled trial.

Of the originally enrolled 190 participants, 154 participated in the 3‐month follow‐up and 106 in the 12‐month follow‐up investigation. Therefore, for this longitudinal observation study, the 190 participants contributed a total of 450 assessments (mean of 1.6 assessments per participant) over the entire study period of 12 months. The recruitment procedure and reasons for loss to follow‐up have been detailed elsewhere.^22^, ^23^ Among others, seven patients had died for various reasons between hospital discharge and the 3‐month follow‐up and 39 patients could not be scheduled at 12 months due to lack of funding. The most frequent reason for missing BDI values at admission was an immediate transfer of patients back to the referring hospitals before they completed questionnaires. Logistical problems with blood sampling were the most frequent reasons for missing D‐dimer values at follow‐up. Several measures were obtained.

### 2.1 Demographic factors

Information on age and sex was taken from medical records.

### 2.2 Indices of cardiac disease severity

These included a history of previous MI, the type of index MI (STEMI or non‐STEMI), left ventricular ejection fraction (LVEF), obtained from angiographic records, and C‐reactive protein (CRP), a marker of the acute inflammatory response during MI and low‐grade systemic chronic inflammation. CRP was measured with an immunoturbidimetric assay (C‐Reactive Protein Gen.3) using the COBAS 8000 c702 module from Roche Diagnostics at the Institute of Clinical Chemistry, Inselspital, Bern University Hospital, Switzerland. For clinical routine, the lab provides CRP values of 20 mg/L and higher as “CRP ≥ 20 mg/L", but no precise concentration. Based on this, and on the prognostic CVD risk with increasing CRP levels, CRP values were categorized as follows: <1 mg/L, 1 to ≤3 mg/L, 3 to ≤10 mg/L, 10 to <20 mg/L, and ≥ 20 mg/L.^24^

### 2.3 Comorbidity

We calculated the Charlson comorbidity index which was categorized by low (index = 1), medium (index =2) or high (index = 3+) 10‐year mortality risk.^25^

### 2.4 Medication

Information on current use of aspirin, other platelet inhibitors (clopidogrel, ticagrelor, and prasugrel), oral anticoagulants (OAC: vitamin K antagonists and direct OAC), statins, antidepressants, and glucocorticoids were collected from medical charts and interviewer‐assessed.

### 2.5 Health behaviors

Body mass index (BMI) was calculated based on self‐disclosed weight and height at admission, and on measured weight and height at both follow‐ups. Patients were categorized in current or nonsmokers. Frequency of physical activity per week “that makes you sweat” was categorized as none, 1–2× or 3–7×. Alcohol consumption was categorized as none, moderate or heavy; the latter based on the number of standard drinks per week (>21 for men, >14 for women).

### 2.6 Cognitive depressive symptoms and history of depression

We assessed cognitive depressive symptoms with the 13‐item self‐rated cognitive depressive symptom subscale of the BDI.^26^ Each item is scored with a value between 0 and 3, referring to the severity of the depressive symptom. The scale yields a total score between 0 and 39. The German version of the 13‐item short form of the BDI is a reliable tool to assess depressive symptoms in patients with CHD and a score ≥ 6 was shown to identify clinically significant depressive symptoms with a sensitivity of 91.7%.^27^ In our sample, Cronbach's α was 0.71 at admission, 0.82 at 3‐months and 0.74 at 12‐months, indicating acceptable internal consistency for the scale. For information on a previous episode of depression, patients were asked, “Have you ever had a depression in your life? (Yes/no)”.

### 2.7 Stress hormones

Norepinephrine was quantified by high‐pressure liquid chromatography in ethylendiamintetraacetic acid (EDTA) plasma using electrochemical detection^28^ at the Laboratory for Stress Monitoring, Gottingen, Germany (inter‐ and intra‐assay coefficients of variation [CV] <10%). Serum cortisol was determined with an electro‐chemiluminescence immunoassay on a Cobas analyzer (Roche Diagnostics, Switzerland) at the Institute of Clinical Chemistry, Inselspital, Bern University Hospital, Switzerland.

### 2.8 Fibrin D‐dimer

Following a strict in‐house protocol to ensure adequate preanalytical conditions, D‐dimer was measured in citrate plasma at the accredited Hemostasis Laboratory, Inselspital, Bern University Hospital, Switzerland. Determination was with a quantitative sandwich enzyme immunoassay (VIDAS‐D‐Dimer, bioMérieux, Geneva, Switzerland) or with a particle‐based immunoturbidimetric assay (Innovance® D‐Dimer; Siemens AG, Munich, Germany). Within‐run CV were 2%–12% and day‐to‐day CV were 2%–3% for both assays. The Vidas® assay is the most clinically validated D‐dimer measurement technique and thus considered the reference commercial quantitative immunoassay.^9^ However, the Innovance® assay has equally good analytical performance as the Vidas® assay^9^ and D‐dimer values are highly correlated (*r* = 0.95) when determined with these two methods.^29^

Data were analyzed with SPSS 26.0 for Windows (SPSS Inc., Chicago, IL) and two‐tailed significance at *p* < .05. We conducted a linear mixed (random effects) regression analysis to examine the covariate‐adjusted longitudinal relation between cognitive depressive symptoms and plasma D‐dimer across three investigations (hospital admission, 3‐month, and 12‐month follow‐ups). With linear mixed model regression an intercept and slope of each participant can be estimated based on the available data for that individual (i.e., even when some points are missing across the three investigations), augmented by the data from the entire sample. We prioritized the continuous BDI score as a predictor variable; however, to test for a relation between clinically significant depressive symptoms and D‐dimer levels, we also used a categorical BDI score of ≥6 versus <6.^27^ Due to a nonnormal distribution, D‐dimer values were log (base 10) transformed prior to analysis. Three outliers with a log‐transformed D‐dimer value greater than three SDs above the sample mean were deleted.

The covariates listed below were considered on an a priori basis as potential confounders of D‐dimer levels.^16^, ^18^ We first modeled univariable associations of cognitive depressive symptoms and each covariate with D‐dimer levels over time. Akaike's information criterion (AIC) showed a better model fit with “time” (i.e., the number of assessments over time) as a log (base 10) transformed variable (AIC = 48.9) instead of a linear variable (AIC = 54.2). This may reflect the fact that D‐dimer rises during acute MI, such that a more rapid decline is expected earlier rather than later after MI.

As a next step, we calculated two multivariable models for cognitive depressive symptoms with covariates entered into models as fixed effects. In Model 1, these covariates were demographics (i.e., age, sex); indices of cardiac disease severity (i.e., index MI, LVEF, CRP, and previous MI); Charlson comorbidity index, depression history, medication use (i.e., aspirin, platelet inhibitors, OAC, statins, antidepressants, and glucocorticoids) and health behaviors (i.e., BMI, smoking, alcohol consumption, and physical activity). In Model 2, we additionally entered stress hormones (i.e., norepinephrine and cortisol levels). Like depressive symptoms, CRP, medication use, health behaviors, and stress hormones were all entered as time varying. Random intercepts were modeled for participants.

Model 2 allowed us to test the extent to which stress hormones potentially mediate the link between cognitive depressive symptoms and D‐dimer. In statistical terms, this would be the case if a significant relation between cognitive depressive symptoms and D‐dimers was no longer significant after stress hormones were added to the equation. Potential effect modification by stress hormones was further tested by entering an interaction term between cognitive depressive symptoms and each stress hormone into Model 2. For significant interactions, we applied the Holmbeck method^30^ to test whether the level of stress hormones (+1 *SD* vs. −1 *SD* from the mean) would moderate the relation between cognitive depressive symptoms and D‐dimer levels.

We computed both between‐ and within‐participants effects of cognitive depressive symptoms on D‐dimer. The between‐participants analysis tests whether patients who are generally (i.e., over the entire study period) higher in cognitive depressive symptoms are also those showing higher D‐dimer levels over the entire study period. The within‐participants analysis tests whether, at individual investigations, a patient who reports a higher level of cognitive depressive symptoms also shows higher D‐dimer, suggesting an association between fluctuations (up or down) in cognitive depressive symptoms and D‐dimer. To obtain within‐participants effects, each patient's personal mean value across time was subtracted from each of his or her individual observation.

## 3 RESULTS

Table 1 shows the characteristics of the study participants at hospital admission and for the two follow‐up investigations. The 190 participants who were included into the study contributed 450 assessments across the three investigations (see legend to Table 1 for the percentage of complete values for each variable). At admission, mean age of participants was 60 years. The majority were men and had rather low comorbidity. Whereas 27.9% of patients reported a history of depressive illness, 13.2% met the cutoff for clinically significant depressive symptoms (BDI score ≥ 6) and 8.4% took antidepressants. The participants who provided assessments at follow‐up had similar time‐invariant characteristics to those who contributed assessments at admission, suggesting that there was little concern for an attrition bias. In terms of time‐variant variables, there was a drop in absolute values of CRP, cortisol, and D‐dimer levels on the one hand, and an increase in more favorable health behaviors with less smoking and greater physical activity on the other. Mean cognitive depressive symptom scores and the prevalence of clinically significant depressive symptoms were similar across the three investigations; yet, the frequency of antidepressant use had almost doubled at 12 months compared to admission. Except for a decrease in the use of platelet inhibitors, the frequency of cardiovascular medication use changed little over time.

**TABLE 1:** Characteristics of study participants at the three investigations

Variables	Admission (*n* = 190)	3‐month follow‐up (*n* = 154)	12‐month follow‐up (*n* = 106)
Time‐invariant			
Age, years, M (*SD*)	59.9 (11.2)	58.7 (10.9)	59.7 (9.9)
Sex, male, *n* (%)	157 (82.6)	130 (84.4)	88 (83.0)
ST‐elevation myocardial infarction, *n* (%)	134 (70.5)	110 (71.4)	74 (69.8)
Left ventricular ejection fraction, %, M (*SD*)	47.6 (11.8)	47.6 (11.8)	48.3 (12.1)
Previous myocardial infarction, *n*, (%)	20 (10.5)	13 (8.4)	9 (8.5)
Charlson comorbidity index			
Low risk, *n* (%)	103 (54.2)	88 (57.1)	54 (50.9)
Medium risk, *n* (%)	49 (25.8	39 (25.3)	30 (28.3)
High risk, *n* (%)	38 (20.0)	27 (17.5)	22 (20.8)
Depression history, *n* (%)	53 (27.9)	45 (29.2)	27 (25.5)
Time‐variant			
C‐reactive protein			
<1 mg/L, *n* (%)	6 (3.2)	62 (40.3)	43 (40.6)
1 to ≤3 mg/L, *n* (%)	14 (7.4)	41 (26.6)	26 (24.5)
3 to ≤10 mg/L, *n* (%)	35 (18.4)	13 (8.4)	12 (11.3)
10 to <20 mg/L, *n* (%)	32 (16.8)	3 (1.9)	0 (0)
≥20 mg/L, *n* (%)	86 (45.3)	1 (0.6)	1 (0.9)
Aspirin, *n* (%)	185 (97.4)	141 (91.6)	96 (90.6)
Platelet inhibitors, *n* (%)	180 (94.7)	133 (86.4)	70 (66.7)
Oral anticoagulants, *n* (%)	21 (11.1)	21 (13.6)	12 (11.3)
Statins, *n* (%)	183 (96.3)	146 (94.8)	100 (94.3)
Antidepressants, *n* (%)	16 (8.4)	15 (9.7)	16 (15.1)
Glucocorticoids, *n* (%)	8 (4.2)	3 (1.9)	2 (1.9)
Body mass index, kg/m^2^, M (*SD*)	28.0 (4.9)	27.4 (4.8)	27.8 (4.9)
Current smoker, *n* (%)	83 (43.7)	20 (13.0)	13 (12.3)
Alcohol consumption			
None	33 (17.4)	37 (24.0)	23 (21.7)
Mild‐to‐moderate	144 (75.8)	108 (70.1)	78 (73.6)
Heavy	10 (5.3)	9 (5.8)	5 (4.7)
Physical activity			
None	88 (46.3)	38 (24.7)	29 (27.4)
1–2×/week	50 (26.3)	20 (13.0)	28 (26.4)
3–7×/week	49 (25.8)	96 (62.3)	49 (46.2)
Norepinephrine, pg/ml, M (*SD*)	565 (360)	667 (313)	662 (275)
Cortisol, nmol/L, M (*SD*)	517 (206)	347 (120)	377 (117)
D‐dimer, ng/ml, M (*SD*)	643 (510)	471 (391)	506 (442)
Depressive symptoms, score, M (*SD*)	2.75 (2.83)	2.46 (3.18)	2.35 (2.65)
Depressive symptoms, score ≥ 6, *n* (%)	25 (13.2)	18 (11.7)	13 (12.3)

*Note:* Due to missing data, some percent values do not add up to 100%. Data for the time‐invariant variables were complete in 100% for age, sex and the Charlson comorbidity index; in 99.1% for index myocardial infarction; in 98.4% for previous myocardial infarction and depression history; and in 96.9% for left ventricular ejection fraction. Data for the time‐variant variables were complete in 99.6 for smoking status; in 99.3% for aspirin, platelet inhibitors, oral anticoagulants, statins, alcohol consumption, and physical activity; in 99.1% for antidepressants and glucocorticoids; in 96.7% for body mass index; in 88.4% for cognitive depressive symptoms; in 83.3% for D‐dimer; in 83.3% for C‐reactive protein; in 82.7% for cortisol; and in 76.7% for noradrenalin.

Univariable associations of between‐participants effects (Table 2) showed a significant decrease in D‐dimer over time (i.e., a significant negative estimate for “time”). Greater cognitive depressive symptom scores, both continuous and categorical, were significantly related to higher D‐dimer levels for the entire study period of 12 months. There were additional significant univariable relations with D‐dimer for higher age, lower LVEF, higher CRP, previous MI, greater comorbidity, glucocorticoid use, less physical activity, higher norepinephrine, and cortisol levels.

**TABLE 2:** Univariable between‐ and within‐participants effects for relations with D‐dimer levels across the entire study period of 12 months

Parameter	Estimate	*SE*	Counts (*n*)
Intercept	2.660***	0.019	372
Time	−0.273***	0.054	372
Age, years	0.042***	0.008	372
Sex, male	−0.016	0.052	372
ST‐elevation MI	0.019	0.043	368
Left ventricular ejection fraction, %	−0.024**	0.008	360
C‐reactive protein	0.055***	0.007	369
Previous myocardial infarction	0.160*	0.066	366
Comorbidity index	0.106***	0.023	372
Depression history	0.030	0.045	366
Aspirin	−0.035	0.058	370
Platelet inhibitors	0.007	0.043	370
Oral anticoagulants	0.003	0.050	370
Statins	0.088	0.068	370
Antidepressants	0.054	0.051	370
Glucocorticoids	0.188*	0.095	370
Body mass index, kg/m^2^	−0.024	0.020	371
Current smoker	0.053	0.031	370
Alcohol consumption	0.008	0.026	369
Physical activity	−0.062***	0.015	369
Norepinephrine (between), pg/ml	0.012*	0.005	335
Cortisol (between), nmol/L	0.039***	0.007	365
Depressive symptoms (between), continuous	0.013*	0.005	337
Depressive symptoms (between), categorical	0.108**	0.040	337
Norepinephrine (within), pg/ml	−0.001	0.007	335
Cortisol (within), nmol/L	0.032***	0.009	365
Depressive symptoms (within), continuous	0.020**	0.007	337

*Note:* ***p < .001; **p < .010; *p < .05 denotes the significance level. Age, left ventricular ejection fraction, and body mass index were entered in 5‐unit steps; norepinephrine and cortisol were entered in 100‐unit steps; cognitive depressive symptom score was entered in 1‐unit steps. Time and D‐dimer were entered as log (base 10) transformed values. All other parameters were entered as categorical variables (see Table 1 for these categories).

The significance of a positive association of both continuous and categorical cognitive depressive symptoms with D‐dimer levels was maintained with adjustment for time, demographic factors, indices of cardiac disease severity, comorbidity, depression history, medication use, health behaviors, and stress hormones (Table 3, Model 1). The results from the fully adjusted Model 2 in Table 3 suggested that stress hormones did not significantly mediate the longitudinal relation between cognitive depressive symptoms and D‐dimer. In addition to cognitive depressive symptoms, higher age, lower LVEF and higher CRP were also independently related to higher D‐dimer; OAC use showed an independent association with lower D‐dimer. In Model 2, patients with clinically significant depressive symptoms (categorical BDI score ≥ 6) had a (antilog) 73 ng/ml higher D‐dimer level than those without (BDI score < 6) when all other variables were held constant (303 ng/ml vs. 230 ng/ml).

**TABLE 3:** Multivariable between‐participants effects for the relations between cognitive depressive symptoms and D‐dimer levels across the entire study period of 12 months

Parameter	Model 1		Model 2	
	Estimate	*SE*	Estimate	*SE*
Intercept	2.331***	0.217	2.337***	0.228
Time	−0.012	0.083	−0.034	0.091
Age, years	0.033***	0.009	0.027**	0.009
Sex, male	0.018	0.050	−0.006	0.052
ST‐elevation myocardial infarction	−0.009	0.046	−0.004	0.046
Left ventricular ejection fraction, %	−0.021*	0.009	−0.021*	0.009
C‐reactive protein	0.049***	0.011	0.052***	0.013
Previous MI	0.016	0.067	0.044	0.070
Comorbidity index	0.050	0.026	0.048	0.025
Depression history	0.019	0.045	0.014	0.046
Aspirin	−0.107*	0.053	−0.099	0.057
Platelet inhibitors	0.001	0.042	0.002	0.044
Oral anticoagulants	−0.149**	0.051	−0.132*	0.054
Statins	0.075	0.061	0.119	0.063
Antidepressants	0.038	0.048	0.078	0.052
Glucocorticoids	0.061	0.085	0.135	0.094
Body mass index, kg/m^2^	−0.017	0.019	−0.032	0.020
Current smoker	−0.006	0.033	−0.026	0.037
Alcohol consumption	0.012	0.023	0.005	0.024
Physical activity	−0.012	0.015	−0.007	0.016
Norepinephrine, pg/ml			0.008	0.005
Cortisol, nmol/L			0.014	0.009
Depressive symptoms, continuous	0.011*	0.005	0.012*	0.005
Depressive symptoms, categorical	0.094*	0.037	0.119**	0.041

*Note:* ***p < .001; **p < .010; *p < .05 denotes the significance level. Age, left ventricular ejection fraction, and body mass index were entered in 5‐unit steps; norepinephrine and cortisol were entered in 100‐unit steps; continuous cognitive depressive symptom score was entered in 1‐unit steps. Time and D‐dimer were entered as log (base 10) transformed values. All other parameters were entered as categorical variables (see Table 1 for these categories). The results for the categorical cognitive depressive symptom score (score ≥ 6) are shown without estimates of the other variables in the models.

Univariable analysis of within‐participants effects (Table 2) showed that an increase in the continuous cognitive depressive symptom score and in cortisol levels, but not in norepinephrine levels, were significantly associated with an increase in D‐dimer levels from one investigation to the next. In the multivariable analysis (Table 4), the significant relation between cognitive depressive symptoms and D‐dimer was maintained with adjustment for time, demographic factors, indices of cardiac disease severity, comorbidity, depression history, medication use and health behaviors (Table 4, Model 1) and, additionally, for within‐participants changes in stress hormones (Table 4, Model 2). However, the relation between cortisol and D‐dimer was no longer significant after adjustment for covariates. The results from Model 2 in Table 4 suggest that changes in stress hormones from one investigation to the next did not mediate the association between fluctuations in cognitive depressive symptoms and D‐dimer levels.

**TABLE 4:** Multivariable within‐participants effects for the relations between cognitive depressive symptoms and D‐dimer levels across the entire study period of 12 months

Parameter	Model 1		Model 2	
	Estimate	*SE*	Estimate	*SE*
Intercept	2.395***	0.214	2.481***	0.222
Time	−0.005	0.084	−0.008	0.092
Age, years	0.031***	0.009	0.029**	0.009
Sex, male	0.019	0.050	0.002	0.052
ST‐elevation myocardial infarction	−0.019	0.046	−0.017	0.046
Left ventricular ejection fraction, %	−0.021*	0.009	−0.023*	0.009
C‐reactive protein	0.050***	0.011	0.053***	0.013
Previous myocardial infarction	0.018	0.067	0.063	0.069
Comorbidity	0.049	0.025	0.046	0.025
Depression history	0.040	0.043	0.035	0.044
Aspirin	−0.104	0.053	−0.089	0.057
Platelet inhibitors	−0.001	0.042	0.004	0.044
Oral anticoagulants	−0.150**	0.052	−0.137*	0.054
Statins	0.073	0.061	0.112	0.064
Antidepressants	0.051	0.048	0.085	0.051
Glucocorticoids	0.070	0.085	0.140	0.094
Body mass index, kg/m^2^	−0.020	0.019	−0.036	0.020
Current smoker	−0.004	0.033	−0.023	0.037
Alcohol consumption	0.016	0.023	0.014	0.024
Physical activity	−0.010	0.016	−0.007	0.017
Norepinephrine (within), pg/ml			0.008	0.006
Cortisol (within), nmol/L			0.017	0.011
Depressive symptoms (within), continuous	0.015*	0.006	0.017*	0.007

*Note:* ***p < .001; **p < .010; *p < .05 denotes the significance level. Age, left ventricular ejection fraction, and body mass index were entered in 5‐unit steps; norepinephrine and cortisol were entered in 100‐unit steps; the continuous depressive symptom score was entered in 1‐unit steps. Time and D‐dimer were entered as log (base 10) transformed values. All other parameters were entered as categorical variables (see Table 1 for these categories).

Next, we tested whether stress hormones would be effect moderators of between‐participants effects. There was a significant cognitive depressive symptom score‐by‐cortisol interaction for D‐dimer levels over time, adjusting for time, demographics, indices of cardiac disease severity, comorbidity, depression history, medication use, health behaviors, norepinephrine levels, and main effects of cortisol levels and cognitive depressive symptoms (estimate [SE] = 0.006 (0.003), *p* < .05). Post hoc analysis revealed that cognitive depressive symptoms were associated with D‐dimer when cortisol levels were high (estimate [SE] = 0.022 (0.007), *p* < .010), but not significantly so when cortisol levels were low (estimate [SE] <0.001 (0.008)). No significant interactions emerged between categorical depressive symptoms and cortisol and between cognitive depressive symptom scores, both continuous and categorical, and norepinephrine levels.

In the within‐participants analysis, there was no significant interaction between continuous cognitive depressive symptoms and both norepinephrine and cortisol levels, adjusting for time, demographics, indices of cardiac disease severity, comorbidity, depression history, medication use, health behaviors, and main effects of both the other stress hormone and cognitive depressive symptoms.

## 4 DISCUSSION

We found a significant longitudinal relation between cognitive depressive symptoms and plasma D‐dimer levels in patients followed for up to 12 months after acute MI. Patients who endorsed more severe cognitive depressive symptoms had greater blood coagulability in the first year following MI than patients with less depressed mood. This relation was independent of a range of factors potentially confounding this relation. In fact, several covariates showed expected associations with D‐dimer levels in univariable and multivariable models. Significant independent associations with elevated D‐dimer emerged for older age, higher CRP and lower LVEF, whereas OAC use was associated with lower D‐dimer.^18^ Moreover, changes in depressive mood severity and plasma D‐dimer concentration from one investigation to the next were significantly related to each other, independent of covariates.

These findings further substantiate the literature on a prothrombotic tendency as a potential mechanism underlying the increased risk of atherothrombotic CVD events in patients with depression. Previous research assessed hypercoagulability in terms of different hemostasis measures, including platelet hyperactivity, clotting factors, global hypercoagulability markers and impaired fibrinolysis.^7^, ^8^ However, unlike our study, previous studies have rarely been performed in depressed patients with established CHD, were cross‐sectional or assessed hemostasis factors at only one point in time.^8^ To illustrate this, in two previous studies of patients with stable CHD, D‐dimer level was unrelated to depressive mood.^31^, ^32^ However, both studies measured D‐dimer only once, and one study did not take potentially confounding variables into account.^31^

Our findings may help to understand why patients with treatment resistant depression,^33^ incident depression^19^ and more severe depression^34^ show a particularly poor prognosis following MI. As a constant severity of cognitive depressive mood over 1 year was related to D‐dimer, hypercoagulability might prevail in the first year after MI in patients who experience no reduction in cognitive depressive symptoms (with or without treatment). Moreover, as a history of depression was not associated with D‐dimer levels over time, our findings substantiate the potential importance of incident cognitive depressive symptoms for hypercoagulability following MI. In addition, the observed gradual relation between cognitive depressive symptoms and D‐dimer suggests that even minimal symptoms of depression may relate to hypercoagulability.

Our findings may have clinical implications. Higher D‐dimer during admission for MI, and when assessed following MI at similar time points as in our study, has been shown to predict recurrent cardiac events.^13^, ^14^, ^15^, ^16^ The 13% of our patients who reached the cut‐off for clinically significant depressive symptoms had a 73 ng/ml higher D‐dimer concentration than those without clinically significant symptoms. This difference is about half that between the top (>273 ng/ml) and bottom (≤112 ng/ml) quartiles of D‐dimer in a large prospective study of patients with acute coronary syndromes.^16^ Compared to patients in the bottom quartile of D‐dimer levels, those in the top quartile showed a 1.5‐fold greater risk of a major coronary event after 6 years and of cardiovascular mortality after 16 years.^16^ Moreover, as fluctuations in cognitive depressive symptoms and D‐dimer showed significant associations, successful treatment of depression could perhaps reduce D‐dimer and cardiac risk within only a few months. This hypothesis deserves to be tested in future studies. To concur, a previous study found decreased platelet activation after 6 months of effective depression treatment with psychotherapy or antidepressants in patients with depressed mood and a history of CVD.^35^ However, as the design of our study does not allow for causal inferences, lowering D‐dimer levels, for example, by improving cardiac function, could also lower depressive mood. Such a bi‐directional view is supported by an increasing literature showing that hemostasis factors are also expressed in the brain and that they may influence brain function and mood.^7^, ^36^, ^37^

We found some evidence for effect modification for cortisol levels. Specifically, there was a significant relation between cognitive depressive symptoms and D‐dimer when cortisol was high for the entire study period, but not when cortisol was low. This observation might reflect cumulative allostatic load referring to the biological and mental burden emerging from adaptation to chronic stress and life events.^38^ Within an allostatic framework, depression might be related to hypercoagulability especially when depressed mood occurs after a stressful experience, which an acute MI is in 70% of cases,^39^ leading to a sustained activation of the hypothalamic–pituitary–adrenal axis. In contrast, cortisol was not a moderator of associations between cognitive depressive symptoms and D‐dimer from one investigation to the next. The reason for this discrepancy could be different dynamics of the relations between cognitive depressive symptoms, cortisol, and D‐dimer over 12 months versus a few months. Another explanation could be single determinations of norepinephrine and cortisol in blood, which provide less reliable measures than 24‐hour urine collection. This methodological shortcoming may also explain why norepinephrine did not modify the relation between cognitive depressive mood and D‐dimer and why stress hormones did not mediate this relation.

The longitudinal design with up to three repetitive investigations and rigorous control of time varying and of potentially confounding variables of D‐dimer values are notable strengths of our study, which also has several limitations worth mentioning. We included patients with high distress during MI who participated in an RCT aimed at preventing psychological sequelae of MI. Although additional adjustment for the intervention group did not change the results (not shown), our findings should not be generalized to the population of patients with acute MI at large. In addition, women were underrepresented in our sample and medical comorbidity was rather low. Venous thromboembolism was not an exclusion criterion, and we could not exclude the possibility that D‐dimer levels were influenced by intercurrent venous thromboembolism during follow‐up, which we did not systematically assess. The item to assess depression history did not ask for a health professional diagnosis of depression and cognitive depressive symptoms following MI were self‐rated. Moreover, for the RCT, we excluded patients with a current severe depressive illness based on clinical judgment. This likely resulted in underrepresentation of patients in the upper range of depressive symptom severity. Whereas the pooled prevalence of depression is 29% in patients with MI,^5^ only 13% met the cut‐off for clinically significant cognitive depressive symptoms in our study; however, the prevalence was similar to other studies when based on cognitive depressive symptoms.^40^ Due to low funding, 39 patients could not be assessed at 12 months.

Taken together, we showed a longitudinal and independent association between cognitive depressive symptoms and plasma D‐dimer levels in patients followed up to 1 year after acute MI. The main finding of our study is consistent with the notion of a prothrombotic tendency in patients with CHD, which can be partially explained by depressed mood. The extent to which hypercoagulability accounts for the increased risk of recurrent cardiac events and poor prognosis in patients with CHD and depression remains to be studied.

## CONFLICT OF INTERESTS

The authors declare that they have no known competing financial interests or personal relationships that could have appeared to influence the work reported in this paper.

## AUTHORS CONTRIBUTION

Roland von Känel: Conceptualization, data curation, formal analysis, funding acquisition, methodology, resources, supervision, writing–original draft, writing–review & editing.

Aju P. Pazhenkottil: Conceptualization, methodology, supervision, validation, writing–review & editing.

Rebecca E. Meister‐Langraf: Conceptualization, data curation, investigation, methodology, project administration, writing–review & editing.

Hansjörg Znoj: Conceptualization, funding acquisition, methodology, supervision, validation, writing–review & editing.

Jean‐Paul Schmid: Conceptualization, funding acquisition, methodology, supervision, validation, writing–review & editing.

Claudia Zuccarella‐Hackl: Conceptualization, methodology, validation, writing–review & editing.

Jürgen Barth: Conceptualization, formal analysis, funding acquisition, methodology, supervision, validation, writing–review & editing.

Ulrich Schnyder: Conceptualization, funding acquisition, methodology, supervision, writing–review & editing.

Mary Princip: Conceptualization, data curation, investigation, methodology, project administration, writing–review & editing.

## Data Availability

The data that support the findings of this study are available on request from the corresponding author. The data are not publicly available due to privacy or ethical restrictions.
